# Psychological Support for Recurrent Pregnancy Loss: A Review of the Current State and Challenges

**DOI:** 10.7759/cureus.112752

**Published:** 2026-07-15

**Authors:** Daichi Inoue

**Affiliations:** 1 Department of Reproductive Medicine, Nishitan ART Clinic Sakae, Nagoya, JPN

**Keywords:** grief, psychological support, recurrent miscarriage, recurrent pregnancy loss, tender loving care, trauma-informed care

## Abstract

Recurrent pregnancy loss (RPL), also referred to as recurrent or habitual miscarriage, is experienced by an estimated 1%-5% of women who conceive and carry a substantial psychological burden, including depression, anxiety, post-traumatic stress symptoms (PTSS), and complicated grief, in addition to its physical impact. Repeated miscarriage and stillbirth act as a cumulative experience of loss, and meta-analytic evidence indicates that they constitute a risk factor for women’s mental health. At the same time, the methodology and evidence base for psychological support specific to RPL remain far less developed than those for etiological investigation and pharmacological treatment. This review synthesizes, with an emphasis on empirical studies, (1) the psychological impact of RPL; (2) the evidence for tender loving care (TLC)/supportive care and psychotherapeutic interventions; (3) the position of psychological support within international guidelines; and (4) the current status and challenges of the support system in Japan. Although there is little scientific basis for the belief that psychological stress is a direct cause of miscarriage, empathic and trauma-informed supportive care, together with structured psychological interventions centered on cognitive behavioral therapy, show a degree of demonstrated effectiveness. The development of a continuous, multidisciplinary support system is required. Positioning psychological support as a *third pillar* of care, alongside etiological investigation and pharmacotherapy, and delivering it continuously with institutional backing is an important future task.

## Introduction and background

Recurrent pregnancy loss (RPL) denotes a condition in which a woman repeatedly experiences miscarriage, stillbirth, or early neonatal death and is consequently unable to obtain a living child [1]. Internationally, it is also termed recurrent miscarriage. For consistency, the term RPL is used throughout this review, and the term recurrent miscarriage is retained only when reporting the specific wording of a cited guideline. Its definition varies across professional societies and countries: the European Society of Human Reproduction and Embryology (ESHRE) defines RPL as two or more pregnancy losses [2], and the Society of Obstetricians and Gynecologists of Canada (SOGC) similarly defines it as two or more losses before 20 weeks of gestation [3]. The condition is not uncommon, affecting an estimated 1%-5% of women who conceive [3].

Beyond its obstetric implications, RPL imposes a substantial psychological burden, as for those affected, it represents a repeated experience of bereavement accompanied by intense emotional distress. Meta-analytic evidence indicates that perinatal loss significantly increases the risk of depression and anxiety [4], and women with RPL specifically show elevated depression, anxiety, and stress relative to controls [5], a higher prevalence of these conditions together with identifiable risk factors [6], and reduced quality of life [7]. Despite the magnitude of this burden, the research base and clinical resources addressing the psychological dimension of RPL have long remained markedly less developed than those devoted to etiological investigation and pharmacological treatment, even as contemporary international guidelines increasingly position psychological support as an integral component of RPL care [2,3].

Against this background, the present review has three objectives: first, to synthesize the current evidence on the psychological impact of RPL; second, to appraise the evidence for interventions that address it, namely tender loving care (TLC)/supportive care, psychotherapeutic and psychosocial interventions, and their positioning within international guidelines; and third, to delineate the current status and challenges of psychological support in Japan. Japan is used here as a case example because it is actively translating international guideline recommendations on psychological support into a publicly funded national system, including national recommendations and dedicated consultation centers; its progress and its persistent gaps - regional disparity, access barriers, and the exclusion of partners - illustrate implementation challenges that are directly relevant to strengthening care models internationally. Through this synthesis, the review aims to inform the development of a continuous, multidisciplinary framework in which psychological support is delivered as a standard element of RPL care.

## Review

Materials and methods

This study is a narrative review. Relevant literature was identified through searches of PubMed (for international literature) and Ichushi-Web (Igaku Chuo Zasshi, for Japanese-language literature), supplemented by the official websites of professional societies and Japanese government agencies for clinical guidelines and national reports. Search terms combined the concepts of RPL and psychological care, including “recurrent pregnancy loss,” “recurrent miscarriage,” “habitual abortion,” “perinatal loss,” and “stillbirth,” each combined with terms such as “psychological,” “mental health,” “grief,” “depression,” “anxiety,” “post-traumatic stress,” “supportive care,” “tender loving care,” “counseling,” and “intervention,” together with the corresponding Japanese terms. The search covered literature from database inception to 2026, with emphasis on peer-reviewed studies, systematic reviews, and meta-analyses from the past decade. Priority was given to (1) empirical studies of the psychological impact of RPL and psychosocial or psychotherapeutic interventions; (2) current international clinical guidelines from the European Society of Human Reproduction and Embryology (ESHRE), the American Society for Reproductive Medicine (ASRM), the Society of Obstetricians and Gynaecologists of Canada (SOGC), the Royal College of Obstetricians and Gynaecologists (RCOG), and the National Institute for Health and Care Excellence (NICE); and (3) Japanese national guidelines, government surveys, and research-group materials on psychological support. Publications outside these languages or without a clear focus on the psychological dimension of pregnancy loss were not included. Because this is a narrative synthesis rather than a systematic review, no formal risk-of-bias assessment or independent quantitative pooling was performed; findings are summarized descriptively, and reported effect sizes are cited as published in the original studies.

Psychological impact of RPL (psychological phenotypes)

Depression, Anxiety, and Stress

A meta-analysis examining the association between perinatal loss and mental health demonstrated a significantly elevated risk of depression and anxiety [4], and increased depression, anxiety, and stress among women with RPL have also been reported [5]. Cross-sectional studies likewise show a high prevalence of depression and anxiety in women with RPL [6]. A systematic review and meta-analysis focused specifically on RPL [5] found that women who had experienced RPL had a higher proportion of moderate-to-severe depression than controls (odds ratio approximately 3.8), with elevated levels of anxiety and stress as well. Furthermore, that study showed that depression, anxiety, and stress were markedly higher in women than in their male partners who had experienced the same RPL, suggesting a gender difference in psychological burden.

At the cross-sectional level, the prevalence of depression and anxiety in women with RPL and associated factors (such as the number of miscarriages) have been reported [6], and women who have experienced recurrent miscarriage have lower quality of life (QOL) and greater psychological distress than controls [7].

Grief, Stigma, and Guilt

Miscarriage and stillbirth are socially disenfranchised losses, which can lead to complicated grief and feelings of isolation [8]. A survey of the Japanese general population has shown that biased attitudes and perceptions toward miscarriage remain among the public [9], and such social perceptions are thought to exacerbate affected individuals’ self-blame and stigma. In fact, the most frequent cause of recurrent miscarriage is the incidental factor of embryonic (fetal) chromosomal abnormality [10,11], and the proportion of miscarriages with chromosomal abnormalities also varies with the number of prior miscarriages. Against this background, Japanese research-group materials position, as the starting point of support, consideration for the self-blame and stigma that affected individuals tend to experience, while premising that grief after miscarriage or stillbirth is a normal reaction [12]. Psychoeducation that shares accurate information with affected individuals - namely, that “most miscarriages cannot be prevented by the mother’s behavior or effort” - is thought to help alleviate self-blame.

Qualitative studies show that stigma impedes psychological recovery and intensifies shame and isolation [13-15]. Brier [8] noted that because grief after miscarriage is a “socially disenfranchised loss,” a lack of understanding from those around the affected person leads to complicated grief and heightened isolation. In the qualitative study by Meaney et al. [13], women who had experienced miscarriage strongly felt a social silence that “this should not be talked about,” and this was directly linked to reinforcement of shame and self-blame.

Bellhouse et al. [14] reported that women who had experienced miscarriage were unable to speak to those around them out of fear of “being thought to be at fault,” resulting in impeded access to support resources. Kersting and Wagner [15] pointed out that such stigma, isolation, and lack of social recognition underlie complicated grief after perinatal loss.

Anxiety in the Subsequent Pregnancy

One psychological characteristic of RPL is that grief over the loss overlaps with intense anxiety and hypervigilance regarding the next pregnancy. Pregnancy after loss is accompanied by chronic tension - “I don’t know when I might lose it again” - and can also affect the formation of the maternal-fetal relationship (attachment to the fetus during pregnancy). The accumulation of such “anticipatory grief” and subsequent-pregnancy anxiety constitutes an important point in support that differs from that following a typical single miscarriage.

Women after miscarriage are prone to chronic vigilance in the subsequent pregnancy [16] and show high anxiety and depression from early pregnancy [17].

Pregnancy after perinatal loss is experienced as a “simultaneous progression of hope and fear,” characterized by excessive vigilance and emotional instability [18]. Blackmore et al. [17] likewise reported that women with a history of pregnancy loss show high levels of anxiety and depression from early pregnancy, with psychological burden persisting throughout the course of the pregnancy.

An integrative review of pregnancy after perinatal loss [18] showed that the subsequent pregnancy is experienced as a “simultaneous progression of hope and fear,” and reported that excessive vigilance, emotional instability, and increased dependence on medical care are particularly characteristic in early pregnancy. This psychological vulnerability is even more pronounced in women with RPL, in whom uncertainty about pregnancy continuation induces strong anxiety.

Moreover, regarding psychosocial interventions for the subsequent pregnancy after perinatal loss, although continuous follow-up and support for the family including the partner are conceivable, a Cochrane systematic review [19] pointed out that high-quality randomized controlled trials (RCTs) are scarce and that the evidence confirming their effectiveness remains limited.

PTSD and Long-Term Psychological Effects

In recent years, it has become clear that RPL and early pregnancy loss increase the risk of developing post-traumatic stress disorder (PTSD). Farren et al. [20] reported that 28%-38% of women met PTSD criteria after miscarriage or ectopic pregnancy [20], and that symptoms persisted even nine months after pregnancy loss [21]. The possibility that psychological distress after miscarriage reaches a clinical level has also been noted [22]. In a longitudinal study by the same research group [21], it became clear that many women continued to have PTSD symptoms even nine months after pregnancy loss, suggesting that the psychological impact in women with RPL is not transient.

Furthermore, Lok and Neugebauer [22] noted that psychological distress after miscarriage can progress beyond a typical grief reaction to clinical-level psychiatric symptoms, and Blackmore et al. [17] showed that a history of prior pregnancy loss is a strong predictor of depression and anxiety in the subsequent pregnancy. These findings indicate that the psychological burden carried by women with RPL can persist not merely as grief but as a trauma reaction and a chronic stress response.

Psychological Distress and Support Needs

The psychological distress of women with RPL is multifaceted, with grief, anxiety, and PTSD symptoms intricately intertwined. In a mixed-methods study of women with RPL [23], it was reported that, in addition to chronic anxiety over “whether the pregnancy can be maintained,” a lack of communication with healthcare providers and uncertainty of information amplified psychological distress. In addition, a network analysis of the post-traumatic stress symptoms that women show in the subsequent pregnancy after pregnancy loss [24] demonstrated mutually reinforcing associations among symptoms, suggesting that the psychological burden in women with RPL may have a structure in which not a single symptom but multiple symptom clusters are maintained in a chained manner.

Theoretical Framework of Psychological Support

Psychological support for RPL generally comprises the following three layers. First is bereavement care for the loss itself, which includes normalization of emotions, memory-making, and the provision of social validation. Second is therapeutic intervention for psychiatric symptoms such as depression, anxiety, and PTSS, which includes cognitive behavioral therapy (CBT) and mindfulness. Third is continuous, supportive monitoring for anxiety during the subsequent pregnancy, centered on so-called tender loving care (TLC)/supportive care. These are not mutually exclusive; it is desirable that they be provided in an integrated manner according to the affected individual’s stage and needs.

Multidisciplinary interventions and evidence

TLC/Supportive Care

TLC is an approach that provides emotional support to women with unexplained recurrent miscarriage through frequent visits and examinations, empathic listening, reassurance, promotion of positive coping, and involvement of the partner. In the classic study regarded as its origin [25], the success rate of the subsequent pregnancy was approximately 86% in the group receiving close monitoring and supportive care, compared with only about 33% in the group not receiving care of the same level; since then, TLC has been widely cited as one of the standard forms of care for unexplained RPL. It should be noted that even after recurrent miscarriage, the live birth rate in the subsequent pregnancy is by no means low, and the prognosis can be estimated according to maternal age and the number of prior miscarriages [26]. Concrete and honest reassurance based on such objective prognostic information is one of the core elements of TLC.

However, it is important to note that this finding is based on an observational study and has not reached confirmation of effectiveness through an RCT. Because TLC is an intervention that carries little harm and may improve the affected individual’s experience, there is also a view positioning it as a useful practice that does not require a rigorous RCT [27]. On the other hand, one should be cautious about overemphasizing the “improvement in pregnancy success rate” through TLC as a biological effect; as described below, there is no established basis for the claim that stress is a direct cause of miscarriage.

The effect of TLC in improving pregnancy outcomes was shown in the classic study [25], and its effectiveness has also been reported in subsequent studies on supportive care. Clifford et al. [28] reported that continuous follow-up and psychological support in a specialized RPL clinic were associated with improved pregnancy outcomes, and Liddell et al. [29] likewise showed a significantly higher live birth rate in the group receiving supportive care.

Furthermore, Quenby et al. [30] showed that “empathic care, continuous monitoring, and reassurance” are central to the support that women with RPL seek, reaffirming the position of supportive care in RPL practice. Regan and Rai [31] likewise stated that in RPL management, psychological support is as important as medical treatment.

A recent mixed-methods study [23] identified the following types of support sought by women with RPL: empathic and non-judgmental attitudes of healthcare providers, continuous follow-up from early pregnancy, access to psychological support (counseling and psychotherapy), family-unit support, including the partner, and transparency of information and accurate explanation of prognosis.

These coincide with the constituent elements of TLC/supportive care and indicate that standardization of RPL support is progressing internationally.

Psychotherapeutic and psychosocial interventions

Non-pharmacological interventions have been shown by meta-analysis to reduce grief and post-traumatic stress symptoms and to enhance perceived social support in parents after perinatal loss [32]. Internet-based CBT (iCBT) is effective in improving grief, depression, and PTSD [33,34]. Mindfulness interventions are also effective in reducing grief and anxiety [35].

A systematic review and meta-analysis of nonpharmacological interventions for parents who had experienced perinatal loss [32] showed, using random-effects models, that such interventions significantly reduced grief (Hedges’ *g* = -0.68; 95% confidence interval (CI) -0.98 to -0.38) and post-traumatic stress symptoms (*g* = -0.69; 95% CI -0.92 to -0.46) and significantly increased perceived social support (*g* = 0.31; 95% CI 0.08-0.55), whereas the pooled effect on parental stress did not reach statistical significance (*g* = -1.34; 95% CI -2.75 to 0.07) and showed very high heterogeneity (*I*² = 96%). These pooled effects, with their CIs and heterogeneity, are summarized in Table 1. The review reported that cognitive-based interventions (such as CBT, mindfulness, and meditation), face-to-face delivery, and individual formats, typically delivered over a small number of sessions, were associated with greater effectiveness, suggesting that structured short-term interventions can be realistic and effective even within limited resources. Intervention content also included psychoeducation about the loss experience, counseling, supportive interventions, mind-body interventions, and interpersonal psychotherapy.

**Table 1:** Pooled effects of nonpharmacological interventions for parents with perinatal loss. Data from Li et al. [32]. Negative values for grief, post-traumatic stress, and parental stress indicate a reduction favoring the intervention; the positive value for perceived social support indicates an increase favoring the intervention. Effects are statistically significant where the 95% CI excludes zero. *The pooled effect on parental stress was not statistically significant and showed very high heterogeneity. CI, confidence interval

Outcome	Trials (participants)	Hedges’ g (95% CI)	Heterogeneity (I^2^)
Grief	10 (976)	-0.68 (-0.98 to -0.38)	79%
Post-traumatic stress symptoms	5 (532)	-0.69 (-0.92 to -0.46)	31%
Parental stress	5 (388)	-1.34 (-2.75 to 0.07)*	96%
Perceived social support	5 (333)	0.31 (0.08 to 0.55)	16%

A prior review integrating psychosocial interventions for parents of perinatal loss [36] also pointed out the importance of bereavement care by midwives and nursing staff. That is, psychological support should be understood not solely as individual therapy by mental-health professionals but as care in a broad sense that includes empathic responses, acknowledgment of the loss, information provision, and continuous engagement by the healthcare providers at the front line of clinical care.

That said, intervention studies are heterogeneous in their target populations (miscarriage/stillbirth/recurrent miscarriage) and outcome measures, and high-quality RCTs specific to RPL remain limited. Meanwhile, a recent mixed-methods study found that women who had experienced two or more miscarriages had significantly greater psychological distress than those who had experienced only one, and cited, as support sought by those affected, compassionate responses from healthcare providers, mental-health support, social support, memory-making, and consideration for the partner [23]. Therefore, psychosocial intervention in RPL must be understood not as narrow “treatment” aimed solely at symptom reduction but as multilayered support that includes acknowledgment of grief, correction of self-blame, modulation of anxiety about the next pregnancy, and support for relationships. Accordingly, in RPL practice as well, for cases in which supportive care alone does not yield sufficient improvement, it is desirable to position structured short-term interventions centered on CBT as a clinical option and to develop a system that can connect individuals to mental-health professionals as needed.

Kersting et al. [34,33] conducted RCTs of iCBT for women after pregnancy loss and showed that grief, depression, and PTSD symptoms improved with moderate effect sizes. This is an important finding demonstrating that structured psychotherapy is effective even in situations where face-to-face support is difficult.

In addition, Dolan et al. [35] reported a systematic review indicating that mindfulness interventions are effective in reducing grief and anxiety, emphasizing the role of attentional control and emotion regulation in psychological recovery after pregnancy loss. Hutti et al. [37] examined healthcare utilization during the pregnancy that follows a perinatal loss, underscoring the additional care burden that affected women carry and its relevance to their psychological adaptation.

Multidisciplinary collaboration and counseling support

Psychological support depends heavily not only on mental-health professionals but also on the quality of everyday communication by obstetricians, nursing staff, and midwives. Empathy, appropriate information provision, and emotional presence promote the affected individual’s adaptation, whereas inappropriate care can hinder recovery. Therefore, it is important to develop training and tools so that even staff who are not psychological specialists can practice a basic supportive attitude.

Partner support

The psychological impact on male partners has long been underestimated, but men also experience grief, isolation, and helplessness in the same way as women [38-40]. Jones et al. [39] pointed out a structural problem whereby men, expected to behave as “the supporting side,” find it difficult to express their own grief. McCreight [40] likewise showed that men’s grief tends to be treated as an “invisible loss” and that they are prone to being left out of support. Moreover, the influence of recurrent miscarriage on the subsequent marital relationship and health has also been examined in the Okazaki Cohort Study in Japan [41], indicating the need to conceive of psychological support not as limited to the individual woman but at the level of the couple. In the Japanese context, several structural factors compound this neglect: perinatal and bereavement care is organized largely within a maternal and child health framework centered on the mother and child, so follow-up, consultation services, and educational materials are directed primarily at the woman; male partners are rarely registered as recipients of care in their own right; male-inclusive information and grief resources are scarce; and workplace leave and prevailing social norms seldom recognize a father’s bereavement [39,40]. Addressing these institutional barriers-by explicitly including partners in consultations and providing male-oriented resources-is necessary to extend bereavement care to the couple as a unit.

A novel approach: compassion-focused therapy

An RCT protocol for internet-delivered compassion-focused therapy (CFT) targeting women with RPL was published in 2026 and is attracting attention as a new intervention model [42]. CFT is considered suitable for women with RPL who have strong self-criticism and guilt, and using it complementarily with conventional CBT is thought to enable more comprehensive psychological support.

Position in international guidelines

ESHRE published the first edition of its RPL guideline in 2017 [43] and revised it in 2022 [2]. The guideline refers to the organization of care and psychological support in the management of RPL, while explicitly stating that there is no evidence that stress is a direct cause of miscarriage. This point is clinically important because many affected individuals attribute the loss to their own stress or behavior; explicitly conveying the guideline-based facts that stress is not a cause and that most losses result from embryonic chromosomal abnormality directly deconstructs the self-blame narrative (“my own stress caused the miscarriage”), reduces guilt, and is therefore itself a therapeutic act rather than mere reassurance. ESHRE further points out that the emotional impact of RPL should be given adequate consideration, that needs for supportive care may differ between men and women, and that appropriate information provision is indispensable. It also states that a specialized RPL clinic should provide not only investigations for etiology but also support and, where possible, treatment, and that the staff involved require appropriate listening skills in addition to experience. In addition, it states that from the outset of care one should candidly share that an effective treatment does not always exist for RPL-indicating that, in a domain of high uncertainty, honest communication is itself part of support.

A similar direction is found in the committee opinion of the ASRM. ASRM [44], after noting that women who have experienced RPL are exposed to the risk of diverse psychological sequelae such as depression, anxiety, post-traumatic stress, guilt, and anger, explicitly states that psychological support is an essential part of miscarriage care and should be offered to all couples who have experienced miscarriage and are planning a future pregnancy. There, healthcare providers are expected to receive the affected individual’s experience as valid, screen for mood disorders and lack of social support, and refer to mental-health professionals as needed. The importance of not blaming patients, as well as of carefully explaining that the most common cause of miscarriage is genetic abnormality of the embryo, is also emphasized.

The SOGC guideline [3] states that RPL requires empathic and trauma-informed care attentive to both physical and emotional aspects, and explicitly notes that affected individuals may need referral to psychological support, counseling, and appropriate mental-health services. In addition, taking into account that a situation in which the optimal course of action is uncertain can itself heighten the affected individual’s anxiety, it emphasizes shared decision-making between provider and patient and effective communication. Furthermore, the JOGC/SOGC guideline strongly recommends providing psychological support, serial follow-up, and early assessment, indicating that psychological support is positioned not as a peripheral element independent of follow-up or diagnostic assessment but at the core of overall management.

Taken together, the major international guidelines all position psychological support for RPL not as an ancillary service but as part of standard care. They commonly emphasize empathic and non-blaming explanation, assessment of grief and anxiety, continuous follow-up, referral to professionals when needed, multidisciplinary collaboration, and shared decision-making. Therefore, psychological support in RPL practice should be understood not as a peripheral response supplementing cases for which medical treatment is not established, but as a clinical practice in itself that alleviates the distress accompanying a highly uncertain condition and supports the affected individual’s decision-making and future outlook.

The Royal College of Obstetricians and Gynaecologists (RCOG) Green-top Guideline No. 17 [45] positions psychological support as an essential element in RPL management and recommends assessment of grief and anxiety, continuous follow-up, and referral to professionals when needed. The National Institute for Health and Care Excellence (NICE) guideline (NG126) [46] likewise explicitly states that emotional care and information provision should be offered as standard care to women after miscarriage. The positions of these major guidelines on psychological support are summarized in Table 2.

**Table 2:** Position of major international guidelines on psychological support in RPL. RPL, recurrent pregnancy loss; ESHRE, European Society of Human Reproduction and Embryology; ASRM, American Society for Reproductive Medicine; SOGC, Society of Obstetricians and Gynaecologists of Canada; RCOG, Royal College of Obstetricians and Gynaecologists; NICE, National Institute for Health and Care Excellence

Guideline (year)	Key statements on psychological support
ESHRE guideline (2022) [2]	Emotional impact should be adequately addressed; supportive-care needs may differ by sex; no evidence that stress causes miscarriage; RPL clinics should offer support and skilled listening
ASRM committee opinion (2026) [44]	Psychological support is essential and should be offered to all couples; screen for mood disorders and lack of social support; refer as needed; avoid blaming patients
SOGC guideline No. 464 (2025) [3]	Empathic, trauma-informed care; refer to psychological support, counseling, and mental-health services; shared decision-making
RCOG Green-top Guideline No. 17 (2023) [45]	Psychological support is an essential element; assess grief and anxiety; provide continuous follow-up and referral
NICE guideline NG126 (2023) [46]	Emotional care and information provision should be offered as standard care after miscarriage

Current status and challenges in Japan

In Japan, the RPL research group of the Japan Agency for Medical Research and Development (AMED) has positioned TLC/supportive care as psychological support for women with RPL in its recommendations and has also advanced the development of awareness and educational materials, including a "Practical Guide to Tender Loving Care (TLC)" for staff who are not psychological specialists. In addition to such research-group-based efforts, at the administrative level the Children and Families Agency positions medical and specialist consultation on infertility and RPL, as well as peer support by those with lived experience, under its “Center for Sexual and Reproductive Health Consultation” program. Standardization and dissemination of support content are also being promoted through the publication of the “Recommendations on the Management of Recurrent Pregnancy Loss 2025” [47] and consultation manuals.

The recommendations state that TLC/supportive care should be provided from the time pregnancy is confirmed, in order to reduce the anxiety and so-called stress of couples with RPL. They further emphasize the importance of securing time and space in which couples with RPL can speak freely, of the entire medical team-including not only physicians but also nurses and midwives-sharing an attitude of listening and empathy, and of explaining clearly in understandable language. In addition, even when a cause cannot be identified, carefully sharing that a prior miscarriage may have been a recurrence of fetal chromosomal abnormality, and that there is a high likelihood of obtaining a living child in the next pregnancy even without pharmacotherapy, is thought to contribute to alleviating self-blame and restoring a future outlook. Furthermore, because grief after miscarriage or stillbirth is a normal reaction while in some cases it can progress to anxiety disorder or depression, a system for referral to mental-health professionals such as psychiatrists as needed is required.

However, the development of systems and recommendations does not in itself guarantee that support reaches those affected. In a national survey of women who had experienced miscarriage or stillbirth (*n* = 618), the proportion who felt distress was 93.0% immediately after the miscarriage or stillbirth, 51.2% at six months, and 32.2% even after more than one year had passed; among those who had experienced stillbirth, 70.0% still carried distress even after more than one year. As reasons for not seeking consultation, in addition to psychological barriers such as “no change can be expected even if I consult” (41.5%) and “I felt reluctant to talk to others” (39.3%), access barriers such as “there was no consultation resource nearby” (34.4%) and “I did not know whom I could consult” (29.5%) were cited [48]. That is, the challenge in Japan lies not only in the presence or absence of support resources themselves but also in the high psychological, informational, and geographical hurdles to reaching support.

Regional disparities in the support system also cannot be overlooked. Whereas 92.9% of prefectures had established a consultation service accepting individual support, 69.3% of ordinary municipalities had not established one, indicating a large bias in the placement of support resources. Furthermore, although 73.3% of partners were perceived to carry strong distress, 41.7% had not consulted anyone, suggesting that partners may be even more prone to isolation than the women themselves [48]. In addition, a survey of the Japanese general population (*n* = 1,219) reported that many people mistakenly assume stress to be a cause of miscarriage, and that 53% of those who had experienced miscarriage harbored guilt and 36% harbored loneliness [9]. These findings indicate that the challenges of RPL support in Japan extend beyond a mere shortage of consultation services or professionals to the insufficiency of partner support, the lack of visibility of consultation resources, and the insufficiency of social awareness efforts to correct stigma and self-responsibility narratives.

In sum, psychological support for RPL in Japan, while founded on recommendations by research groups and system development by the administration, still faces the challenges of regional disparity, access barriers, and a narrow scope of support recipients. Going forward, it is necessary to make medical institutions function as the entry point for support and to build a multilayered support system connecting to the administration, mental-health professionals, and peer support, while in parallel expanding the support framework to include not only the woman herself but also the partner, and advancing improvement of social understanding regarding miscarriage and RPL.

Challenges and future directions

Several challenges must be addressed for psychological support in RPL to mature into standard care. First, high-quality randomized controlled trials of psychological interventions specific to RPL are lacking, and verification with standardized populations, measures, and outcomes is needed. Second, research and support-which tend to be biased toward women-must be extended to male partners and to the couple unit. Third, a framework is required that conceptually distinguishes the “psychological effect” of TLC from its “effect on pregnancy outcomes” and evaluates the former with person-centered outcomes such as QOL, distress, and satisfaction. Fourth, it is desirable to incorporate, as a standard element of support, psychoeducation that dispels the misconception that stress causes miscarriage.

## Conclusions

RPL is not only an obstetric condition but a repeated bereavement that imposes a serious and frequently persistent psychological burden on those affected. The central message of this review is that, although stress is not a direct cause of miscarriage, the psychological suffering that accompanies RPL is real, clinically meaningful, and amenable to support. Empathic, trauma-informed, supportive care and structured short-term interventions centered on CBT can meaningfully alleviate this suffering, particularly when they are delivered continuously and are extended beyond the woman to her partner and the couple. Psychological support should therefore be understood not as an optional adjunct offered only when medical treatment is unavailable, but as a third pillar of RPL care that stands alongside etiological investigation and pharmacotherapy. Translating this principle into practice calls for concrete, implementable steps within standard obstetric care. Services should consider the following: (1) embedding brief, validated psychological screening at the time of RPL diagnosis and again in early subsequent pregnancy; (2) establishing clear referral pathways from RPL clinics to mental-health professionals for those who screen positive; (3) training obstetric, nursing, and midwifery staff in tender loving care and trauma-informed communication so that supportive care is delivered by the whole team; (4) routinely including partners in consultations and providing male-inclusive information and grief resources; (5) standardizing psychoeducation that corrects the misconception that stress causes miscarriage; (6) reducing regional disparities in access through consultation-center networks and telehealth-delivered follow-up; and (7) securing the institutional and funding structures that make continuous, multidisciplinary follow-up the default rather than the exception. Ultimately, attending to the psychological dimension of RPL is inseparable from providing good clinical care.
